# Supplementary figures and images for: Multilocus molecular systematics of the circumtropical reef-fish genus Abudefduf (Pomacentridae): history, geography and ecology of speciation
Source: PeerJ. 2018 Aug 14;6:e5357. doi: 10.7717/peerj.5357 (PMC6097498; doi:10.7717/peerj.5357)

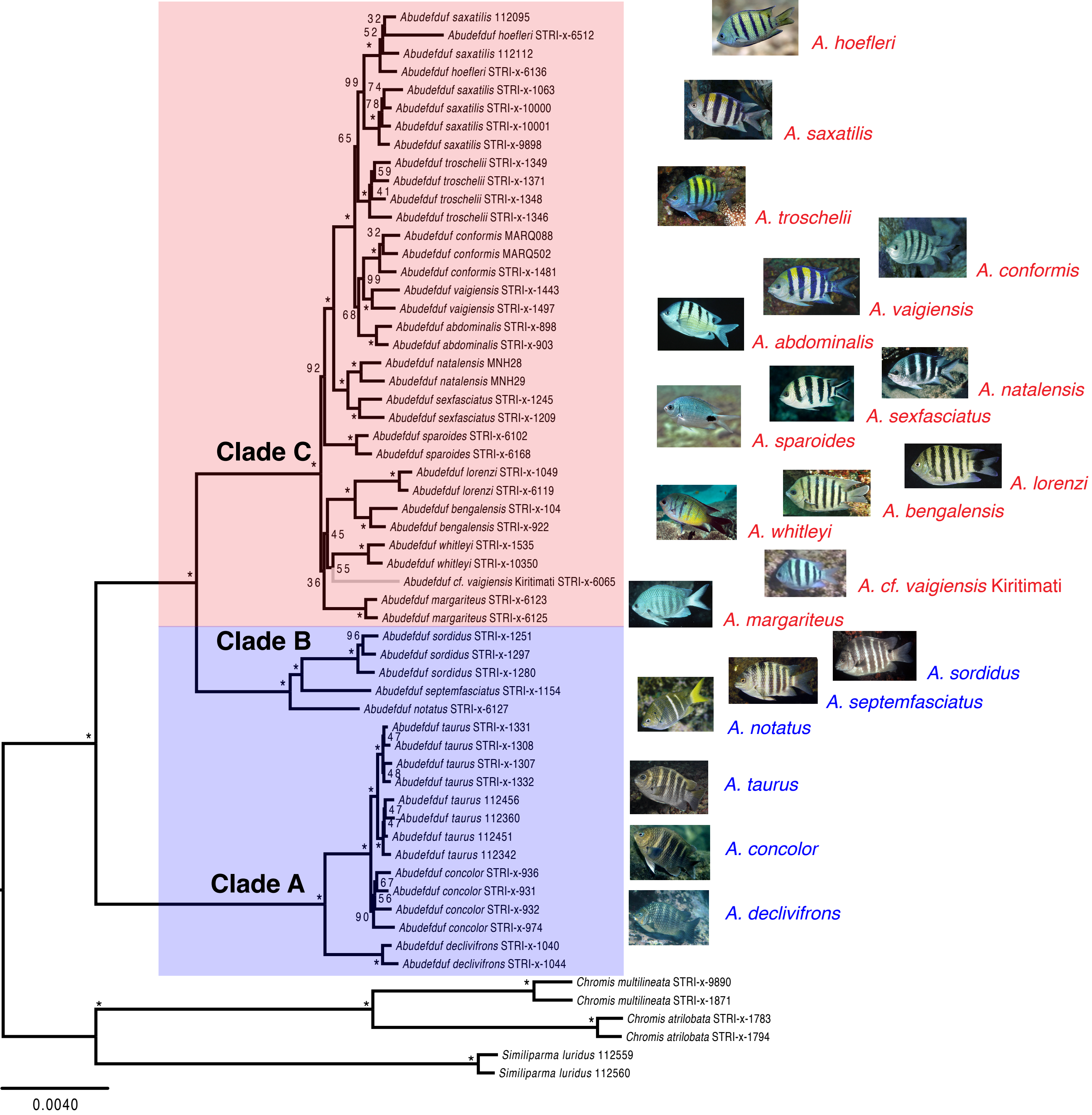

Supplement: Figure S1 — A maximum likelihood phylogenetic of Abudefduf generated from partitioned analysis of 361 ultraconserved element (UCE) loci. An optimal partitioning strategy was implemented (Lanfear et al., 2014; Lanfear et al., 2012) . Each partition was modeled under the General Time Reversible (GTR) model of sequence evolution with Gamma distributed rate variation (G). Bootstrap support values are indicated with an asterisk (*) if equal to 100. The three lineages of Abudefduf are indicated (Clade A, B and C). Planktivorous lineages are highlighted and named in red with benthivorous lineages highlighted and named in blue. The tree is rooted by pomacentrid outgroups of the genera Chromis and Similiparma. Appended to each leaf is the identifier of the particular sample examined. Photo credits: A. hoefleri S. Floeter, A. saxatilis DRR, A. troschelii GRA, A. conformis J. Randall, A. vaigiensis GRA, A. abdominalis GRA, A. sparoides GRA, A. sexfasciatus J. Greenfield (CC BY), A. natalensis J. Randall, A. whitleyi GRA, A. bengalensis G. Edgar (CC BY), A. lorenzi GRA, A. margariteus GRA, A. cf. vaigiensis J. Earle, A. notatus GRA, A. septemfasciatus GRA, A. sordidus GRA, A. taurus DRR, A. concolor GRA, A. declivifrons GRA. [file peerj-06-5357-s003.pdf]

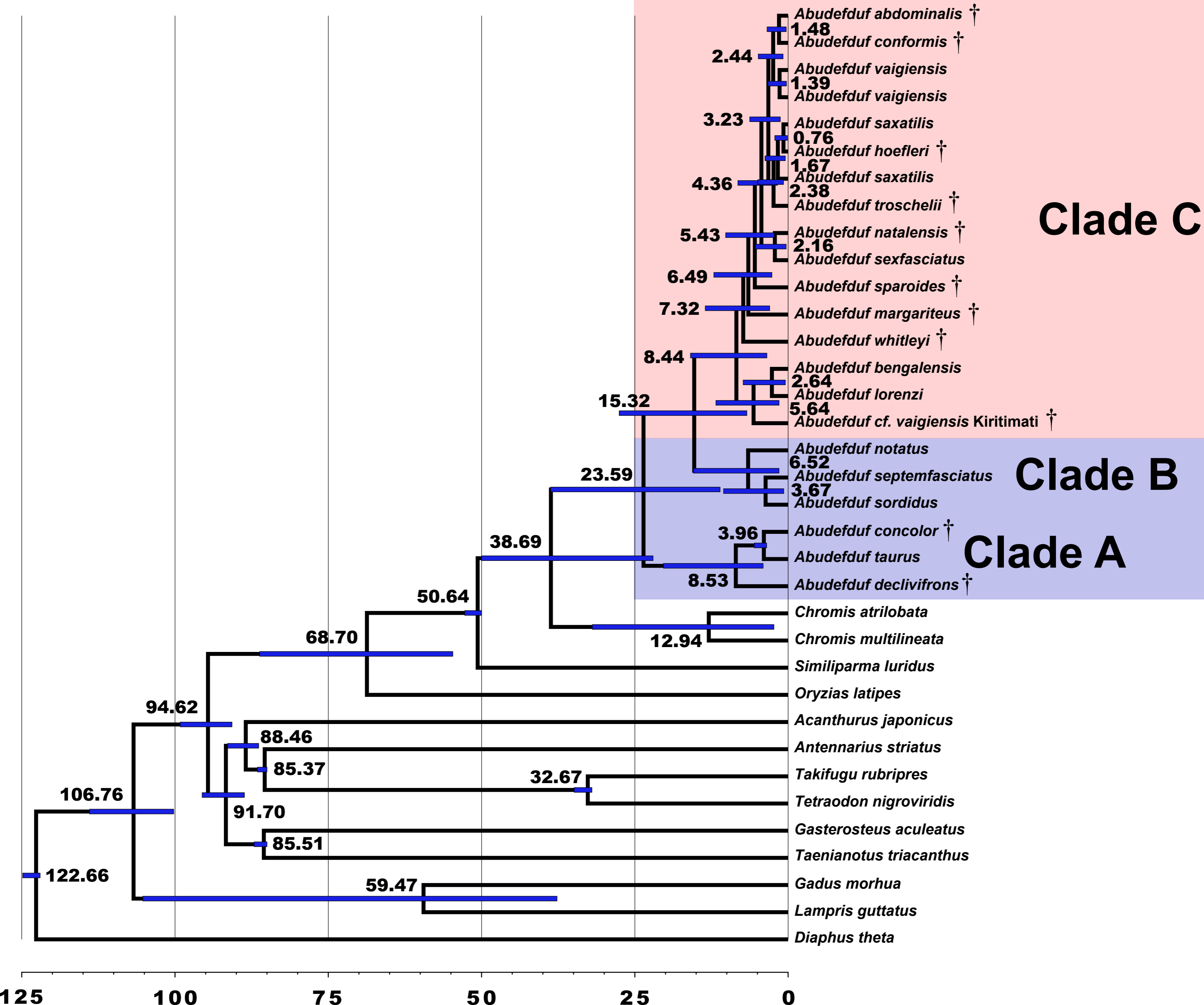

Supplement: Figure S2 — Time-calibrated phylogenetic tree of Abudefduf generated from 137 ultraconserved element (UCE) loci modeled under a single partition with the General Time Reversible (GTR) model with Gamma distributed rate variation (Γ). Fossil calibration points are described in Table S2. Nodes are labeled with posterior medians of divergence time estimates with blue bars indicating 95% highest posterior density. The major trophic guilds are indicated by shading of red for planktivores and blue for benthivores. Regional endemics are indicated by a dagger (†). Individual samples included in this analysis are indicated in Table S1. [file peerj-06-5357-s004.pdf]

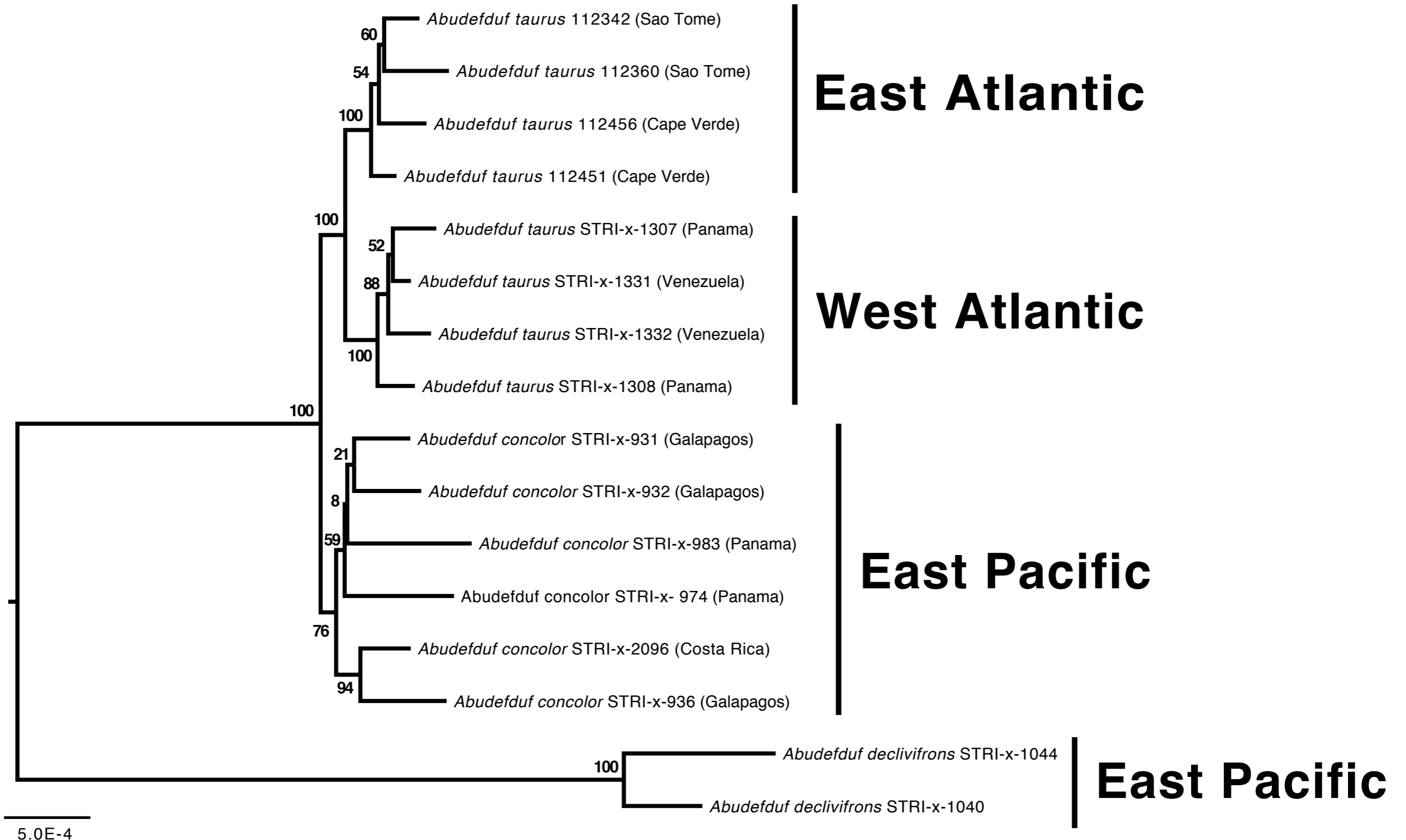

Supplement: Figure S3 — A maximum likelihood phylogenetic tree of Abudefduf taurus and A. concolor samples examined for this study. The tree was generated from 118 ultraconserved element (UCE) loci modeled under three partitions as defined by PartitionFinder (see Methods). Each partition was modeled with the General Time Reversible (GTR) model with Gamma distributed rate variation (Γ) and bootstrapping was conducted to assess confidence, with bootstrap values shown at nodes. Individual tissue identifiers and the general geographic collection area are appended to taxon names. For A. taurus, the clear geographic division between the East and West Atlantic is indicated. The other two species examined are both from the East Pacific which is also indicated on the figure. [file peerj-06-5357-s005.pdf]

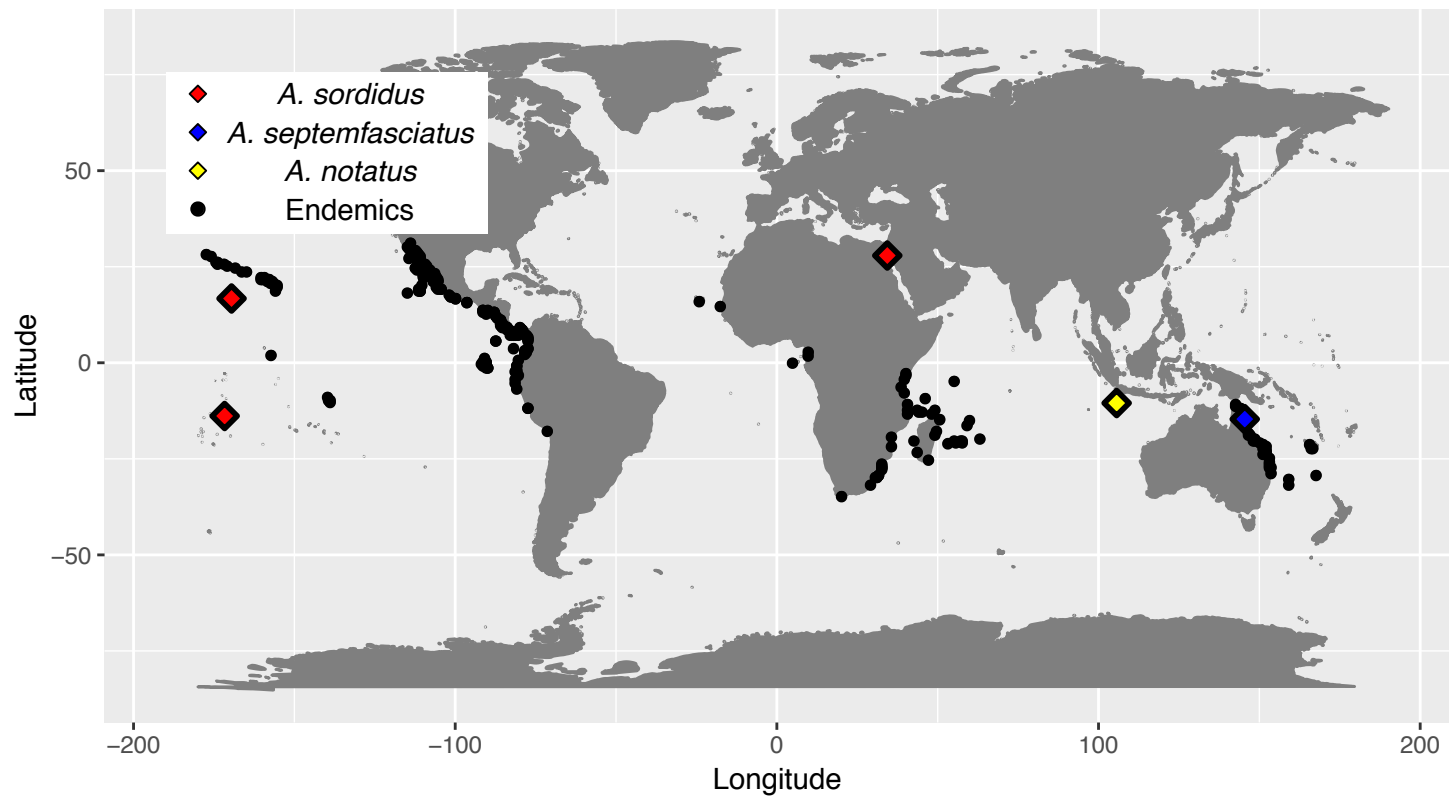

Supplement: Figure S4 — Comparison of sampling locations of the Abudefduf sordidus Clade, “Clade B” to the distribution of regional endemic Abudefduf species. Clade B sampling points are plotted color-coded to species as indicated in the key, while endemic distribution points are plotted from data points as described in Range Map Methods.docx. [file peerj-06-5357-s006.pdf]

Distribution Map of *A. saxatilis* and *A. vaigiensis* Clades

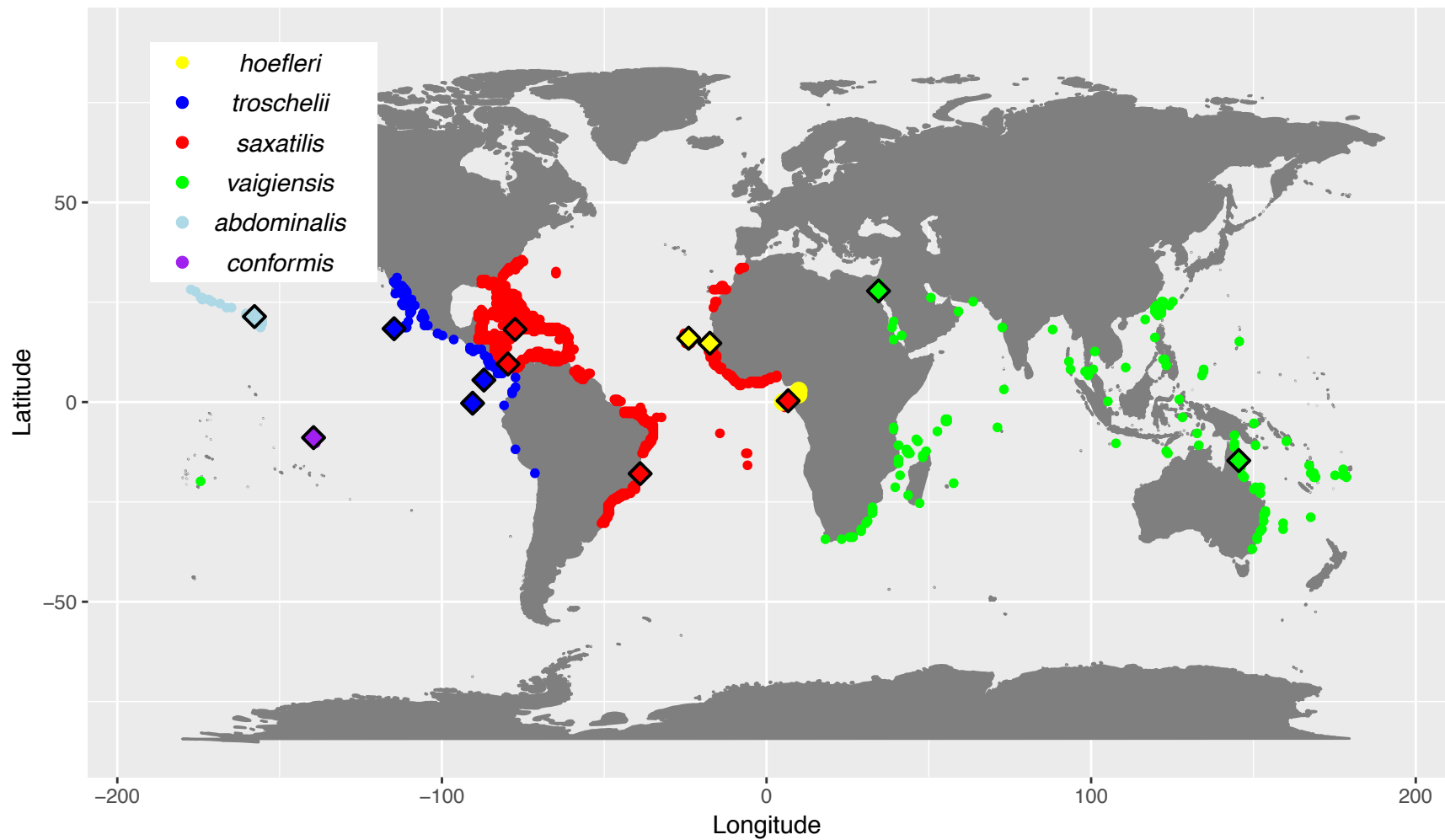

Supplement: Supplemental Information 1 [file peerj-06-5357-s007.pdf]

Distribution Map of *A. sexfasciatus*, *A. sparoides*, *A. whitleyi* and *A. margariteus*

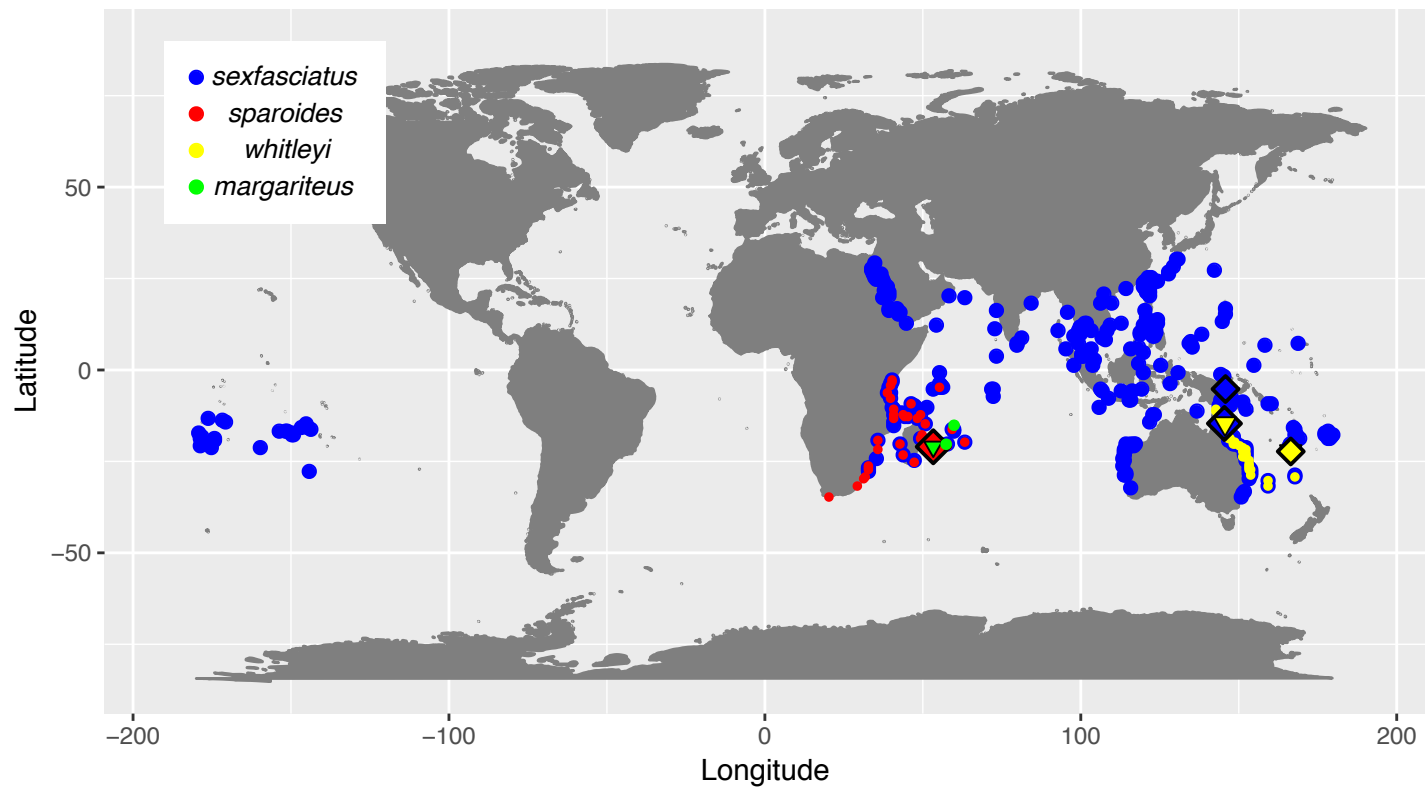

Supplement: Supplemental Information 2 [file peerj-06-5357-s008.pdf]

Distribution Map of *A. bengalensis*, *A. lorenzi*, *A. natalensis*, and *A. cf. vaigiensis* Kiritimati

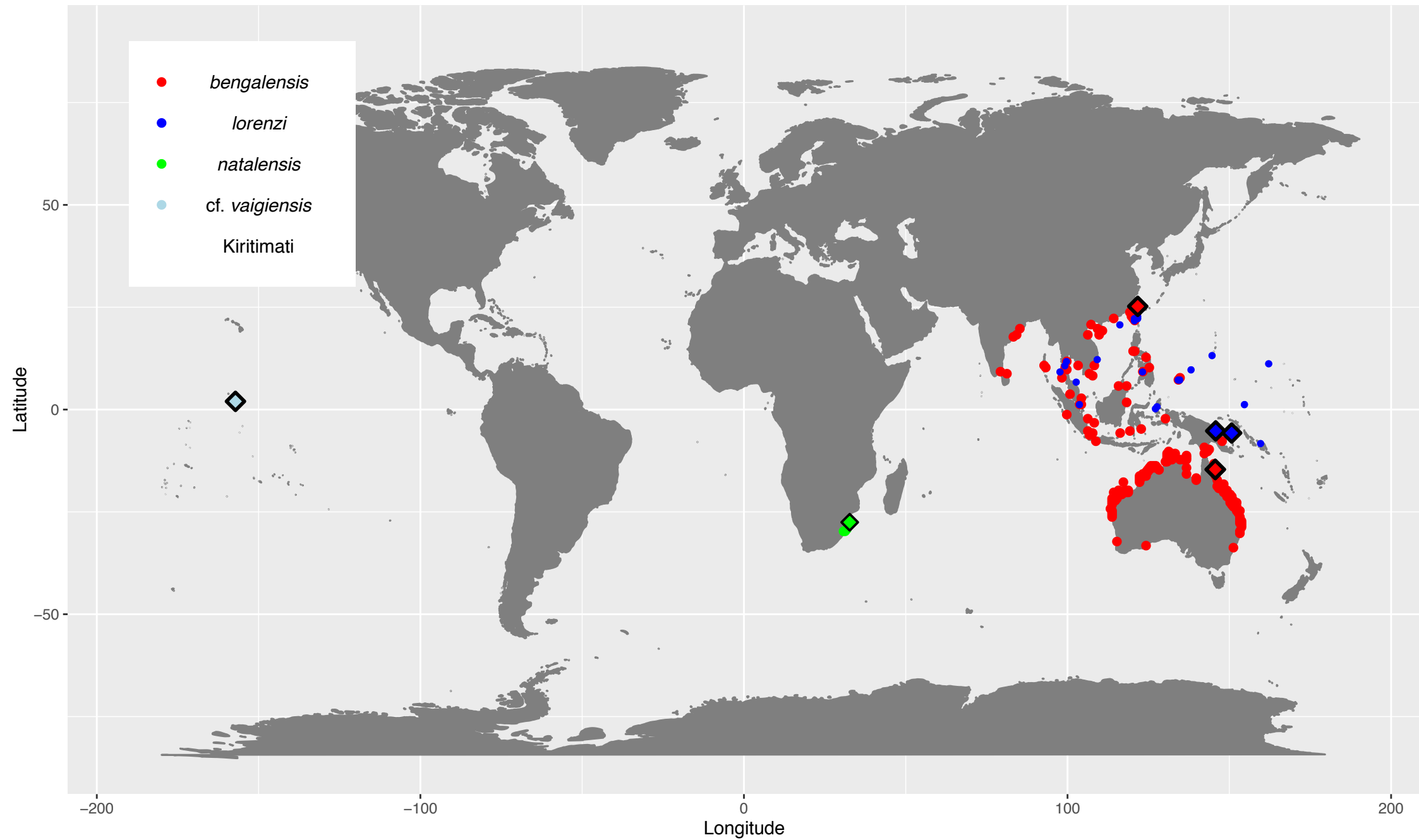

Supplement: Supplemental Information 3 [file peerj-06-5357-s009.pdf]

Distribution Map of *A. notatus* Clade

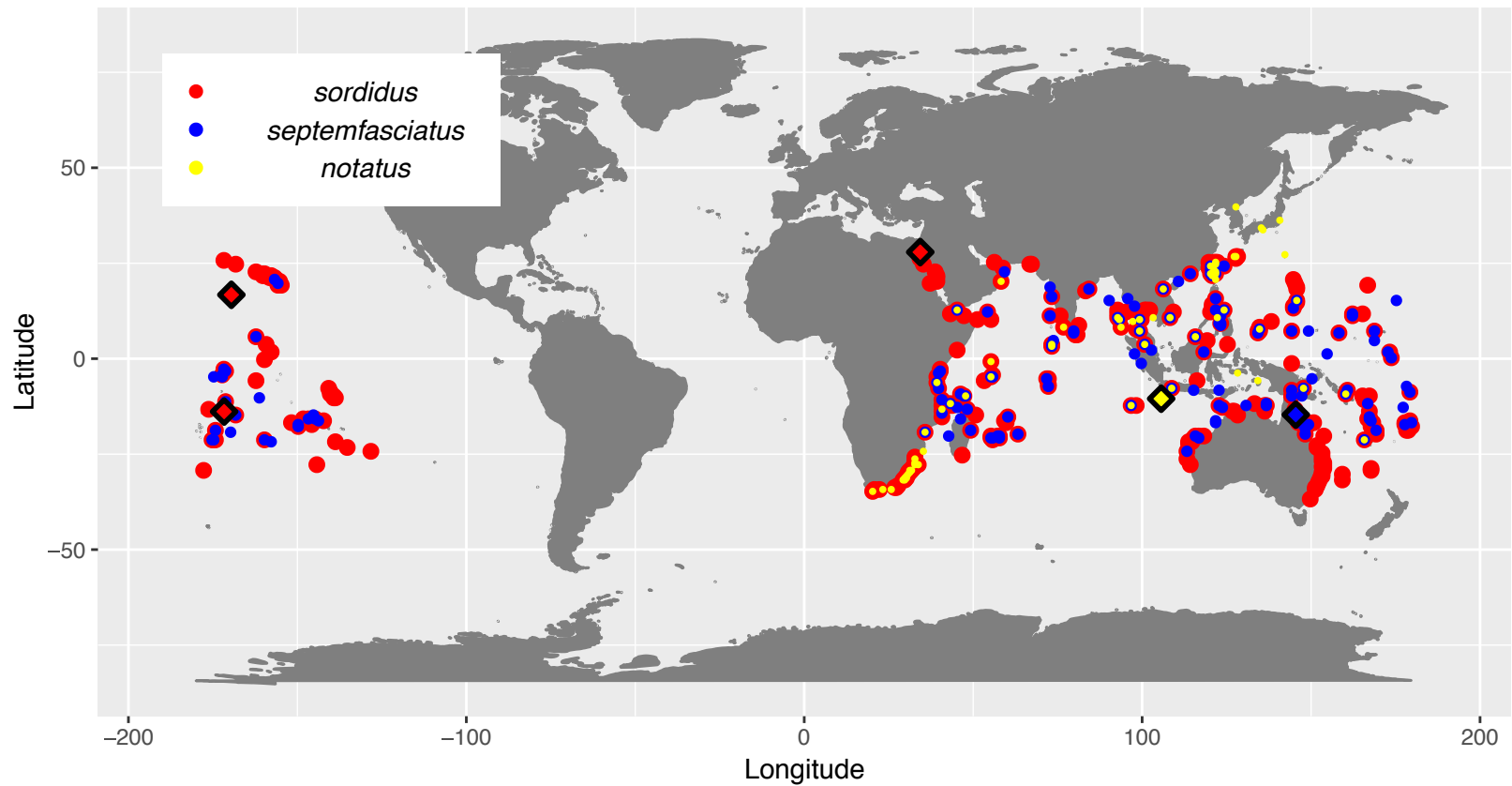

Supplement: Supplemental Information 4 [file peerj-06-5357-s010.pdf]

Distribution Map of *A. taurus* Clade

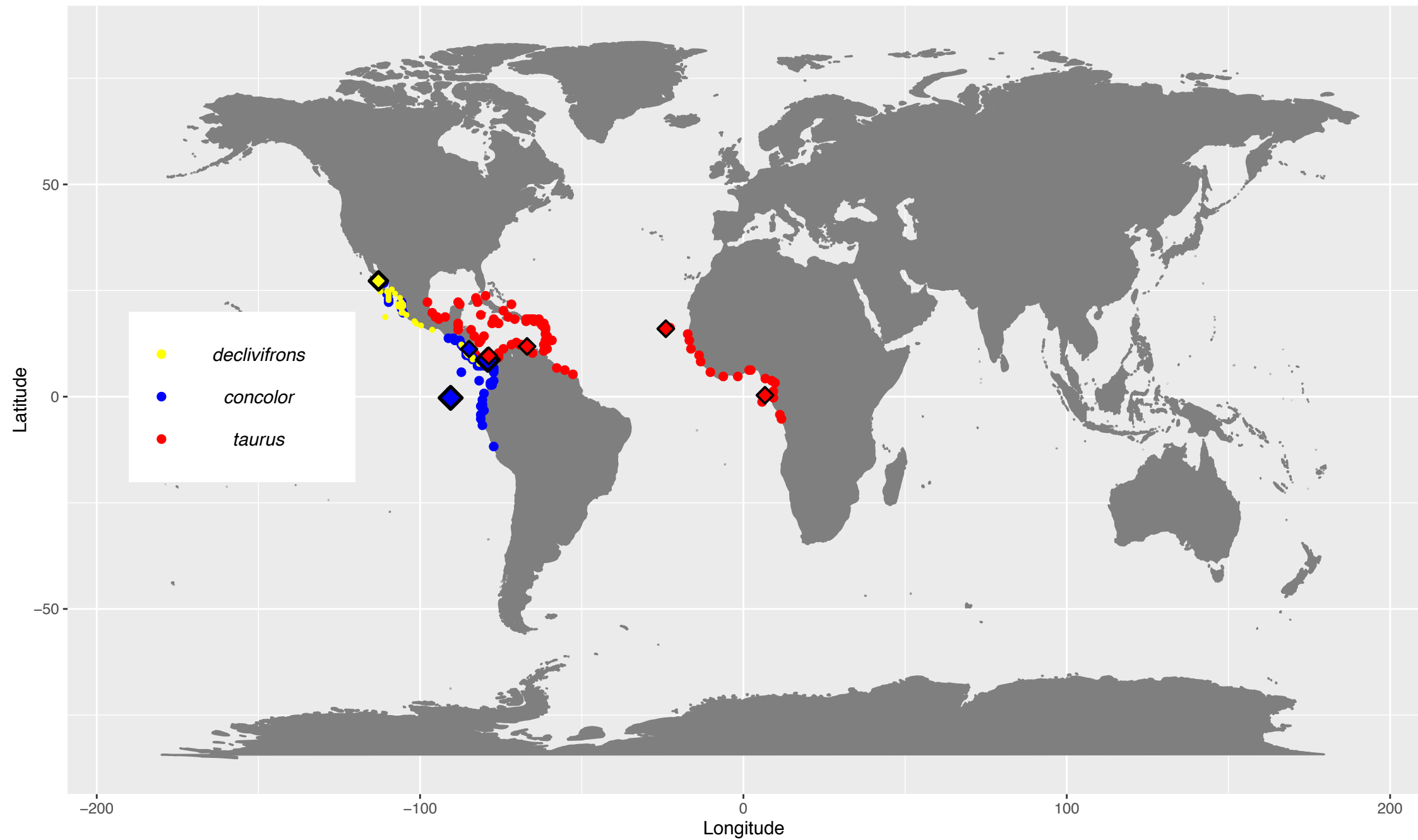

Supplement: Supplemental Information 5 [file peerj-06-5357-s011.pdf]
