# Supplementary material for: Multilocus molecular systematics of the circumtropical reef-fish genus Abudefduf (Pomacentridae): history, geography and ecology of speciation
Source: PeerJ. 2018 Aug 14;6:e5357. doi: 10.7717/peerj.5357 (PMC6097498; doi:10.7717/peerj.5357)
Supplement: Supplemental Information 6 [file peerj-06-5357-s012.docx]

**Range Map Methodology**

Range data for each described species was downloaded from Aquamaps on August 8, 2016 (Kaschner et al. 2015). For regional the species *Abudefduf* *hoefleri, A. margariteus*, and *A. vaigiensis* “Kiritimati” no data were available from Aquamaps. We downloaded distribution data from museum specimens from FishNet2 (http://www.fishnet2.net/) on January 13, 2018 for *A. hoefleri* and *A. margariteus*. No data for distribution is available for *A. vaigiensis* “Kiritimati” besides the collection location for the sample examined in this study.

Each coordinate set from Aquamaps is associated with a probability of occurrence = 1.00, except *A. saxatilis* which was reduced to 0.90 to filter out occurrences outside known range. Data were uploaded in R version 3.3.0 (R Development Core Team 2016) and plotted on a world map with ggmap version 2.6.1 (Kahle and Wickham 2013)and ggplot version 2.2.1 (Wickham 2009). For each species the Aquamap distribution points are plotted as a single color, with collection locations bordered in black.

**Bibliography**

Kahle D, Wickham H. 2013. ggmap: Spatial Visualization with ggplot2. R J. 5:144–161.

Kaschner K, Kesner-Reyes K, Garilao C, Rius-Barile J, Rees T, Froes R. 2015. AquaMaps: Predicted range maps for aquatic species. AquaMaps. [accessed 2016 Aug 8]. www.aquamaps.org.

R Development Core Team. 2016. R: A language and environment for statistical computing. Vienna, Austria: R Foundation for Statistical Computing.

Wickham H. 2009. ggplot2: Elegant Graphics for Data Analysis. New York: Springer-Verlag.
