# Supplementary material for: Multilocus molecular systematics of the circumtropical reef-fish genus Abudefduf (Pomacentridae): history, geography and ecology of speciation
Source: PeerJ. 2018 Aug 14;6:e5357. doi: 10.7717/peerj.5357 (PMC6097498; doi:10.7717/peerj.5357)
Supplement: Appendix S1 [file peerj-06-5357-s014.docx]

**Appendix 1**

M.A. Campbell, D.R. Robertson, M.I. Vargas, G.R. Allen & W.O. McMillan

**“**Multilocus molecular systematics of the circumtropical reef-fish genus *Abudefduf* (Pomacentridae): history, geography and ecology of speciation”

*PeerJ*

**Section A1.1 Appendix Summary**

In this manuscript we put forth evidence from our multilocus analysis that supports the hypothesis that benthivorous species of *Abudefduf* are less prone to generating regional endemics, have few cryptic species and are less species-rich than planktivorous congeners. Our multilocus sampling of one of the two benthivore clades (Clade A) is sufficient in geographic terms and lends support to the idea that benthivores lack high levels of cryptic diversity and local endemism. However, our sampling is geographically insufficient to show whether or not that is the case with the second benthivore clade, Clade B, which includes two species with large, overlapping Indo-Central Pacific geographic ranges. For those two Clade B species we did not sample many parts of their ranges, particularly a variety of sites that contain local endemics of planktivore Clade C (Supplemental Figure S4). For *Abudefduf septemfasciatus* we obtained sequences from only one location (Figure AI-1). With *A. sordidus*, we sampled three, well-separated sites at the limits of its range (Figure A1-1). One of the *A. sordidus* sampling locations was shared with a local-endemic planktivore; however, there is no evidence of species-level differentiation across large distances in *A. sordidus*.

To further test this hypothesis about the lack of cryptic diversity in Clade B we examined data available in GenBank for many more samples collected more broadly across the ranges of both *A. septemfasciatus* and *A. sordidus*.

**Section A1.2 Methods**

From the existing assemblies of *A. septemfasciatus* and *A. sordidus* (included in the Data Supplement) we searched for mitochondrial contigs with the Basic Local Alignment Search Tool (BLAST) (Altschul et al., 1997). More specifically, the mitochondrial cytochrome b (cytb) sequence of AP006016.1, the *A. vaigiensis* mitochondrial genome sequence, was used as a reference and sequences searched against it with the *blastn* algorithm using BLAST 2.4.0. From *A. septemfasciatus* STRI-x-1154 1016 base pairs (bp) of cytb alignment were identified. From *A. sordidus* specimens STRI-x-1251, STRI-x-1280, and STRI-x1297, 1161 bp, 634 bp, and 166 bp of cytb sequence data were retrieved from the assemblies (Table A1-1). These sequences were combined with data from GenBank for *A. septemfasciatus* and *A. sordidus* (Table A1-2). Five additional sequences were included for rooting and are described in Table A1-2.

Nucleotide sequences were aligned with MAFFT 7.130b (Katoh et al., 2002; Katoh and Standley, 2013; Katoh and Toh, 2008) then a maximum likelihood (ML) tree was generated with Randomized Axelerated Maximum Likelihood (RAxML) 8.0.19 (Stamatakis, 2014, 2006; Stamatakis and Ott, 2008). We modeled a single data partition with the General Time Reversible (GTR) model of nucleotide evolution incorporating gamma-distributed rate variation (-m GTRGAMMA) with 1,000 bootstrap replicates to assess confidence at nodes.

**Section A1.3 Results**

The alignment of cytb sequences has 84 sequences, is 1,179 characters in length, contains 3.88% gaps or missing data and has 212 alignment patterns (available in Data Supplement). For *A. sordidus* the phylogenetic tree generated from the Genbank data includes samples from five well-separated parts of its range, including two that have local-endemic planktivores: Johnston island in the central Pacific (*A. abdominalis*, see Range Map 1 and Figure A1-1) and the southwest Indian Ocean, with three local-endemic planktivores (*A*. *margariteus, A*. *sparoides* and *A*. *natalensis*, see Range Maps 2 and 3, Supplemental Figure S4 and Figure A1-1). There is little evidence of structure in the mitochondrial clades of *A. sordidus* and nothing associated with geography: specimens from both Taiwan and the southwest Indian Ocean are scattered all through the *A. sordidus* section of the cytb tree in Figure A1-2. For *A*. s*eptemfasciatus* we obtained samples from four parts of its range, including two with local-endemic planktivores: the Coral Sea (*A. whitleyi* see Range Map 2) and the southwest Indian Ocean (3 local endemics, see above) (Figure A1-2). While there is evidence of geographic structure in the *A. septemfasciatus* tree (Figure A1-2) this does not appear to be associated with local endemism as all four major lineages that were well sampled are present at Taiwan, the population at Aceh (eastern Indian Ocean) includes three of those lineages, and the Coral Sea hosts two of them (Figure A1-2). We conclude that this more extensive mitochondrial dataset indicates that both species in benthivore Clade B lack local endemics, cryptic or non-cryptic, and supports the hypothesis that benthivorous *Abudefduf* are markedly less diverse and have distinctly fewer local endemics than planktivorous congeners.

**Section A1.4 Figures**

**
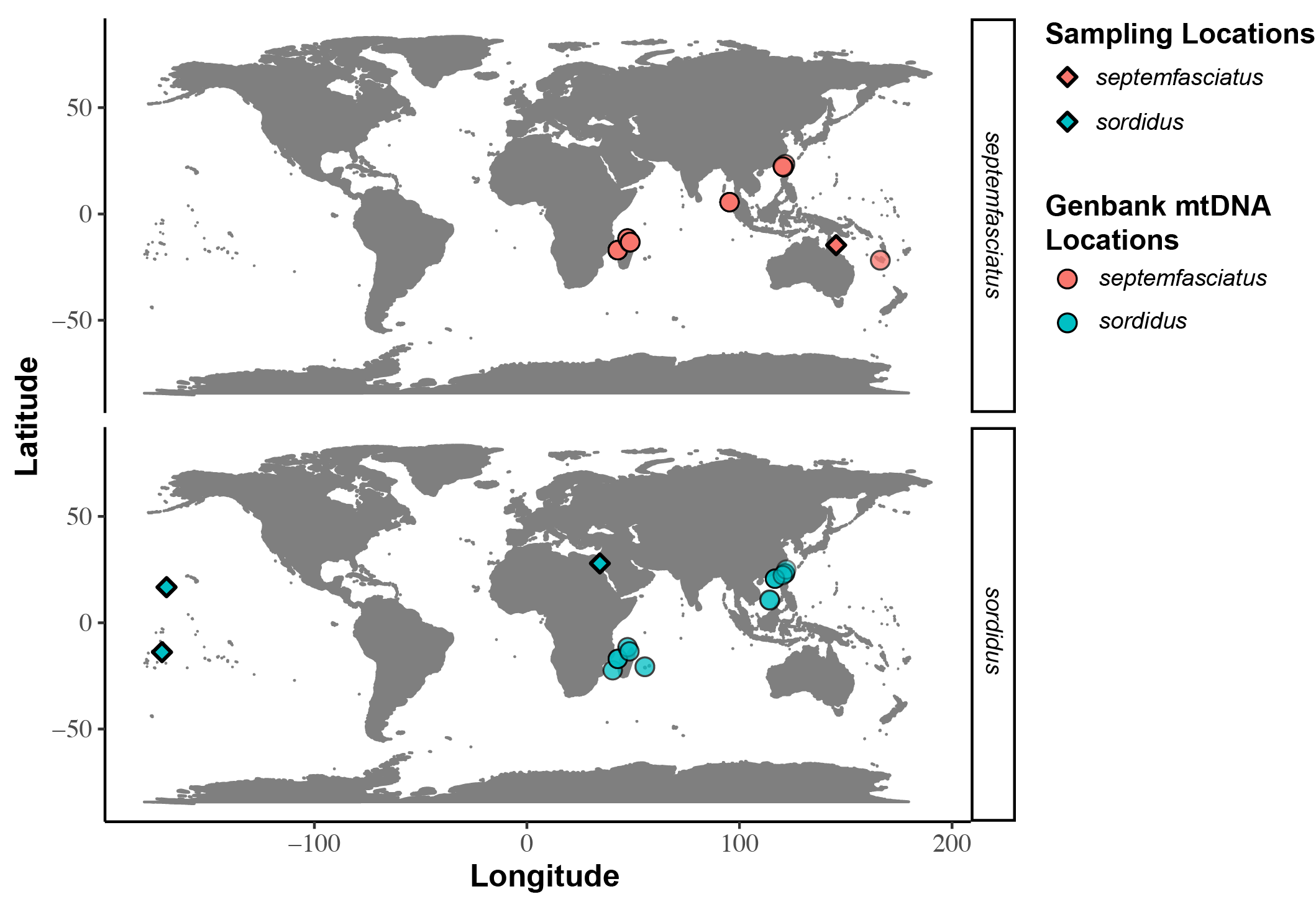
**

**Figure AI-1.** Geographic origins of mitochondrial DNA sequence data.

**
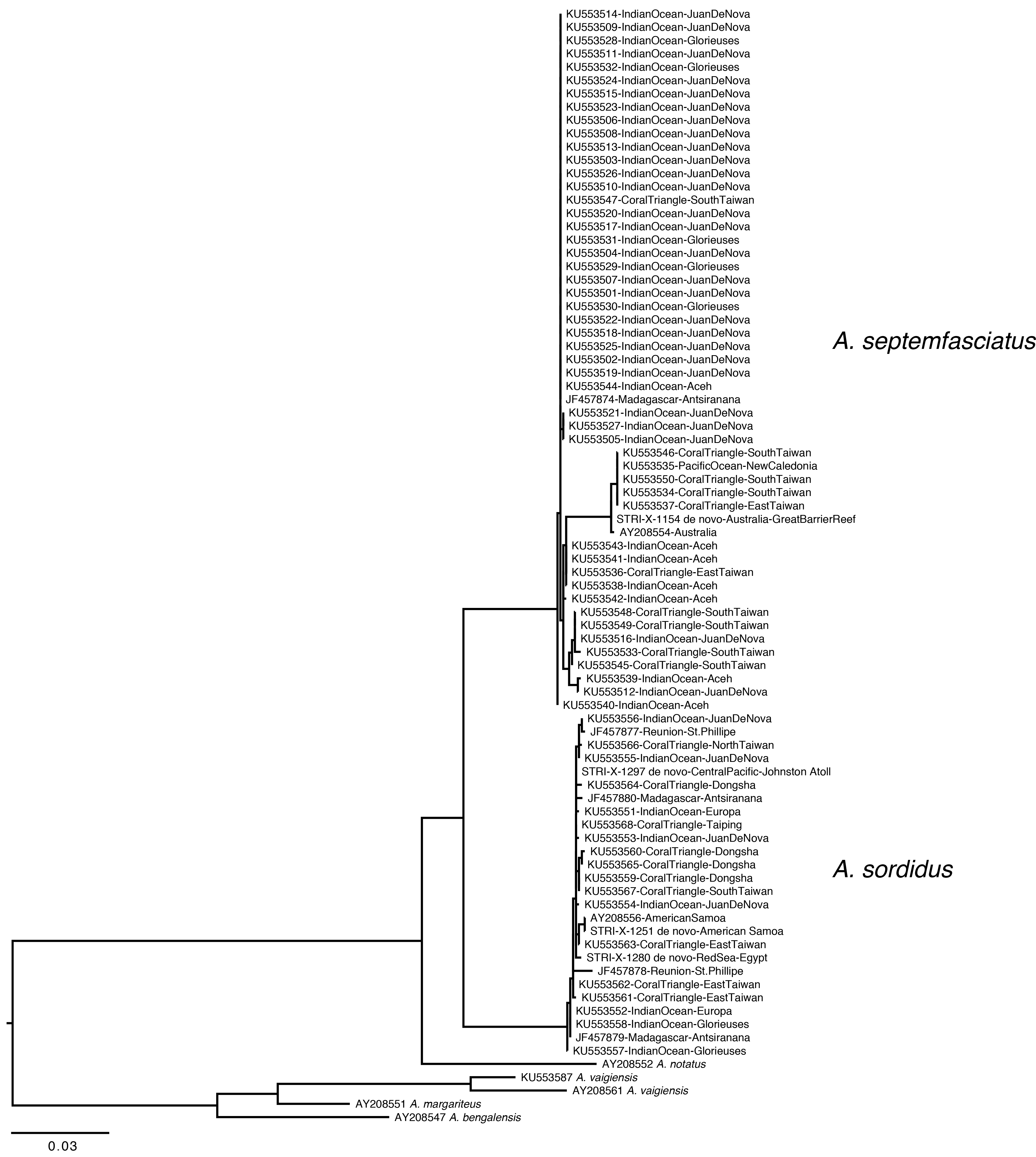
**

**Figure AI-2.** Maximum likelihood phylogenetic tree of Clade B *Abudefduf* mitochondrial data. Mitochondrial cytochrome b (cytb) sequences from GenBank were combined with data from *de novo* assemblies from individuals sequenced in this study. Nucleotide sequence evolution was modeled under the General Time Reversible (GTR) model of sequence evolution with gamma-distributed rate variation (Γ) with 1,000 bootstrap replicates to evaluate confidence of nodes. Bootstrap values are not presented in the figure and the tree file is available in the Data Supplement. Sequences from *de novo* assemblies generated in this study are named with individual labels as in Supplemental Table S1 and as “de novo” with geographic information appended.

**Section A1.5 Tables**

**Table A1-1.** Mitochondrial cytochrome b (cytb) sequences retrieved from *de novo* assemblies in this study from *A. septemfasciatus* and *A. sordidus*. The species, Smithsonian Tropical Research Institute (STRI) identifier, contig name from assembly, geographic region with specific locality and coordinates are provided.

| **Species** | **Identifier** | **Contig** | **Region** | **Locality** | **Latitude** | **Longitude** |
| --- | --- | --- | --- | --- | --- | --- |
|  |  |  |  |  |  |  |
| *septemfasciatus* | STRI-X-1154 | NODE_89_length_1588_cov_6.187657 | Australia | Great Barrier Reef | -14.67 | 145.46 |
| *sordidus* | STRI-X-1251 | NODE_57_length_4059_cov_7.375216 | none | American Samoa | -13.85 | -171.68 |
| *sordidus* | STRI-X-1280 | NODE_566_length_490_cov_3.751020 | Red Sea | Egypt | 34.33 | 27.91 |
| *sordidus* | STRI-X-1297 | NODE_1150_length_405_cov_2.079012 | Central Pacific | Johnston Atoll | 16.73 | -169.54 |

**Table A1-2.** Species of *Abudefduf* and accession of mitochondrial cytochrome b (cytb) sequences retrieved from GenBank. For *A. septemfasciatus* and *A. sordidus* the general geographic region with specific locality and coordinates are given if known.

| **Species** | **Accession** | **Region** | **Locality** | **Latitude** | **Longitude** |
| --- | --- | --- | --- | --- | --- |
|  |  |  |  |  |  |
| *septemfasciatus* | AY208554 | Australia | none | none | none |
| *septemfasciatus* | KU553545 | Coral Triangle | South Taiwan | 21.98 | 120.75 |
| *septemfasciatus* | KU553546 | Coral Triangle | South Taiwan | 21.98 | 120.75 |
| *septemfasciatus* | KU553547 | Coral Triangle | South Taiwan | 21.98 | 120.75 |
| *septemfasciatus* | KU553548 | Coral Triangle | South Taiwan | 21.98 | 120.75 |
| *septemfasciatus* | KU553533 | Coral Triangle | South Taiwan | 22.35 | 120.37 |
| *septemfasciatus* | KU553534 | Coral Triangle | South Taiwan | 22.35 | 120.37 |
| *septemfasciatus* | KU553549 | Coral Triangle | South Taiwan | 22.35 | 120.37 |
| *septemfasciatus* | KU553550 | Coral Triangle | South Taiwan | 22.35 | 120.37 |
| *septemfasciatus* | KU553536 | Coral Triangle | East Taiwan | 23.24 | 121.41 |
| *septemfasciatus* | KU553537 | Coral Triangle | East Taiwan | 23.48 | 121.51 |
| *septemfasciatus* | KU553501 | Indian Ocean | Juan De Nova | -17.03 | 42.72 |
| *septemfasciatus* | KU553502 | Indian Ocean | Juan De Nova | -17.03 | 42.72 |
| *septemfasciatus* | KU553503 | Indian Ocean | Juan De Nova | -17.03 | 42.72 |
| *septemfasciatus* | KU553504 | Indian Ocean | Juan De Nova | -17.03 | 42.72 |
| *septemfasciatus* | KU553505 | Indian Ocean | Juan De Nova | -17.03 | 42.72 |
| *septemfasciatus* | KU553506 | Indian Ocean | Juan De Nova | -17.03 | 42.72 |
| *septemfasciatus* | KU553507 | Indian Ocean | Juan De Nova | -17.03 | 42.72 |
| *septemfasciatus* | KU553508 | Indian Ocean | Juan De Nova | -17.03 | 42.72 |
| *septemfasciatus* | KU553509 | Indian Ocean | Juan De Nova | -17.03 | 42.72 |
| *septemfasciatus* | KU553510 | Indian Ocean | Juan De Nova | -17.03 | 42.72 |
| *septemfasciatus* | KU553511 | Indian Ocean | Juan De Nova | -17.03 | 42.72 |
| *septemfasciatus* | KU553512 | Indian Ocean | Juan De Nova | -17.03 | 42.72 |
| *septemfasciatus* | KU553513 | Indian Ocean | Juan De Nova | -17.03 | 42.72 |
| *septemfasciatus* | KU553514 | Indian Ocean | Juan De Nova | -17.03 | 42.72 |
| *septemfasciatus* | KU553515 | Indian Ocean | Juan De Nova | -17.03 | 42.72 |
| *septemfasciatus* | KU553516 | Indian Ocean | Juan De Nova | -17.03 | 42.72 |
| *septemfasciatus* | KU553517 | Indian Ocean | Juan De Nova | -17.03 | 42.72 |
| *septemfasciatus* | KU553518 | Indian Ocean | Juan De Nova | -17.03 | 42.72 |
| *septemfasciatus* | KU553519 | Indian Ocean | Juan De Nova | -17.03 | 42.72 |
| *septemfasciatus* | KU553520 | Indian Ocean | Juan De Nova | -17.03 | 42.72 |
| *septemfasciatus* | KU553521 | Indian Ocean | Juan De Nova | -17.03 | 42.72 |
| *septemfasciatus* | KU553522 | Indian Ocean | Juan De Nova | -17.03 | 42.72 |
| *septemfasciatus* | KU553523 | Indian Ocean | Juan De Nova | -17.03 | 42.72 |
| *septemfasciatus* | KU553524 | Indian Ocean | Juan De Nova | -17.03 | 42.72 |
| *septemfasciatus* | KU553525 | Indian Ocean | Juan De Nova | -17.03 | 42.72 |
| *septemfasciatus* | KU553526 | Indian Ocean | Juan De Nova | -17.03 | 42.72 |
| *septemfasciatus* | KU553527 | Indian Ocean | Juan De Nova | -17.03 | 42.72 |
| *septemfasciatus* | KU553528 | Indian Ocean | Glorieuses | -11.53 | 47.33 |
| *septemfasciatus* | KU553529 | Indian Ocean | Glorieuses | -11.53 | 47.33 |
| *septemfasciatus* | KU553530 | Indian Ocean | Glorieuses | -11.53 | 47.33 |
| *septemfasciatus* | KU553531 | Indian Ocean | Glorieuses | -11.53 | 47.33 |
| *septemfasciatus* | KU553532 | Indian Ocean | Glorieuses | -11.53 | 47.33 |
| *septemfasciatus* | KU553538 | Indian Ocean | Aceh | 5.55 | 95.32 |
| *septemfasciatus* | KU553539 | Indian Ocean | Aceh | 5.55 | 95.32 |
| *septemfasciatus* | KU553540 | Indian Ocean | Aceh | 5.55 | 95.32 |
| *septemfasciatus* | KU553541 | Indian Ocean | Aceh | 5.55 | 95.32 |
| *septemfasciatus* | KU553542 | Indian Ocean | Aceh | 5.55 | 95.32 |
| *septemfasciatus* | KU553543 | Indian Ocean | Aceh | 5.55 | 95.32 |
| *septemfasciatus* | KU553544 | Indian Ocean | Aceh | 5.55 | 95.32 |
| *septemfasciatus* | JF457874 | Madagascar | Antsiranana | -13.4772 | 48.239 |
| *septemfasciatus* | KU553535 | Pacific Ocean | New Caledonia | -20.93 | 165.4 |
| *sordidus* | AY208556 | none | American Samoa | none | none |
| *sordidus* | KU553568 | Coral Triangle | Taiping | 10.38 | 114.37 |
| *sordidus* | KU553559 | Coral Triangle | Dongsha | 20.72 | 116.7 |
| *sordidus* | KU553560 | Coral Triangle | Dongsha | 20.72 | 116.7 |
| *sordidus* | KU553564 | Coral Triangle | Dongsha | 20.72 | 116.7 |
| *sordidus* | KU553565 | Coral Triangle | Dongsha | 20.72 | 116.7 |
| *sordidus* | KU553567 | Coral Triangle | South Taiwan | 22.35 | 120.37 |
| *sordidus* | KU553561 | Coral Triangle | East Taiwan | 23.24 | 121.41 |
| *sordidus* | KU553562 | Coral Triangle | East Taiwan | 23.24 | 121.41 |
| *sordidus* | KU553563 | Coral Triangle | East Taiwan | 23.24 | 121.41 |
| *sordidus* | KU553566 | Coral Triangle | North Taiwan | 25.02 | 122 |
| *sordidus* | KU553551 | Indian Ocean | Europa | -22.37 | 40.37 |
| *sordidus* | KU553552 | Indian Ocean | Europa | -22.37 | 40.37 |
| *sordidus* | KU553553 | Indian Ocean | Juan De Nova | -17.03 | 42.72 |
| *sordidus* | KU553554 | Indian Ocean | Juan De Nova | -17.03 | 42.72 |
| *sordidus* | KU553555 | Indian Ocean | Juan De Nova | -17.03 | 42.72 |
| *sordidus* | KU553556 | Indian Ocean | Juan De Nova | -17.03 | 42.72 |
| *sordidus* | KU553557 | Indian Ocean | Glorieuses | -11.53 | 47.33 |
| *sordidus* | KU553558 | Indian Ocean | Glorieuses | -11.53 | 47.33 |
| *sordidus* | JF457879 | Madagascar | Antsiranana | -13.4833 | 48.233 |
| *sordidus* | JF457880 | Madagascar | Antsiranana | -13.4833 | 48.233 |
| *sordidus* | JF457877 | Reunion | St. Phillipe | -21.365 | 55.7678 |
| *sordidus* | JF457878 | Reunion | St. Phillipe | -21.365 | 55.7678 |
|  |  |  |  |  |  |
| Outgroup Sequences | |  |  |  |  |
|  |  |  |  |  |  |
| *bengalensis* | AY208547 |  |  |  |  |
| *margariteus* | AY208551 |  |  |  |  |
| *notatus* | AY208552 |  |  |  |  |
| *vaigiensis* | KU5535857 |  |  |  |  |
| *vaigiensis* | AY208561 |  |  |  |  |
|  |  |  |  |  |  |
|  |  |  |  |  |  |

**Section A1.6 Bibliography**

Altschul, S.F., Madden, T.L., Schäffer, A.A., Zhang, J., Zhang, Z., Miller, W., Lipman, D.J., 1997. Gapped BLAST and PSI-BLAST: a new generation of protein database search programs. Nucleic Acids Research 25, 3389–3402. https://doi.org/10.1093/nar/25.17.3389

Katoh, K., Misawa, K., Kuma, K., Miyata, T., 2002. MAFFT: a novel method for rapid multiple sequence alignment based on fast Fourier transform. Nucleic Acids Res. 30, 3059–3066. https://doi.org/10.1093/nar/gkf436

Katoh, K., Standley, D.M., 2013. MAFFT multiple sequence alignment software version 7: improvements in performance and usability. Molecular Biology and Evolution 30, 772–780. https://doi.org/10.1093/molbev/mst010

Katoh, K., Toh, H., 2008. Recent developments in the MAFFT multiple sequence alignment program. Brief. Bioinform. 9, 286–298. https://doi.org/10.1093/bib/bbn013

Stamatakis, A., 2014. RAxML version 8: a tool for phylogenetic analysis and post-analysis of large phylogenies. Bioinformatics 30, 1312–1313. https://doi.org/10.1093/bioinformatics/btu033

Stamatakis, A., 2006. RAxML-VI-HPC: maximum likelihood-based phylogenetic analyses with thousands of taxa and mixed models. Bioinformatics 22, 2688–2690. https://doi.org/10.1093/bioinformatics/btl446

Stamatakis, A., Ott, M., 2008. Efficient computation of the phylogenetic likelihood function on multi-gene alignments and multi-core architectures. Philosophical Transactions of the Royal Society B: Biological Sciences 363, 3977–3984. https://doi.org/10.1098/rstb.2008.0163
